# Experience-based optimal foraging on planktonic prey in Baikal seals

**DOI:** 10.1186/s40462-025-00593-y

**Published:** 2025-09-30

**Authors:** Yuuki Y. Watanabe, Eugene A. Baranov, Nobuyuki Miyazaki

**Affiliations:** 1 Research Center for Integrative Evolutionary Science, The Graduate University for Advanced Studies, SOKENDAI, Hayama, Kanagawa, 240-0193 Japan; 2 Baikal Seal Aquarium, Irkutsk, 664005 Russia; 3 Atmosphere and Ocean Research Institute, The University of Tokyo, Kashiwa, Chiba, 277-8564 Japan

**Keywords:** Biologging, Foraging behaviour, Lake Baikal, Optimal foraging theory

## Abstract

**Background:**

Understanding how predatory animals efficiently locate prey with limited knowledge of its location is challenging. Optimal foraging theory suggests that animals improve their food intake through experience-based adjustments of search patterns. For example, animals feeding on clustered prey may repeatedly search successful areas and move farther away when unsuccessful (the ‘win-stay, lose-shift’ strategy). A related concept, area-restricted search, predicts that animals initially search broadly and then switch to a more localized, tortuous search upon finding clustered prey. However, few studies have empirically supported these predictions for large aquatic predators due to difficulties in recording their foraging success on known prey species.

**Methods:**

We used biologging techniques to record the fine-scale foraging behaviour of Baikal seals in Lake Baikal, which hunt tiny, clustered, planktonic amphipods at high rates. We reconstructed their three-dimensional movement paths during dives and estimated the timing of prey capture events based on video-validated body acceleration data.

**Results:**

Seals moved shorter horizontal distances and exhibited greater directional changes after more successful dives, supporting the ‘win-stay, lose-shift’ strategy. Consistent with area-restricted search, successful foraging dives led to decreased speed and increased tortuosity in the horizontal plane.

**Conclusions:**

These findings suggest that experience-based behavioural adjustments at a dive-to-dive scale are crucial for Baikal seals—and possibly other large aquatic predators—to maintain high foraging rates. Furthermore, they illustrate how an exceptionally high predator-prey body mass ratio (> 500,000) for a single-prey-feeding (non-filter-feeding) predator is maintained in the unique Lake Baikal ecosystem.

**Supplementary Information:**

The online version contains supplementary material available at 10.1186/s40462-025-00593-y.

## Background

Predatory animals generally have limited knowledge of the spaciotemporal distribution of prey. Natural selection has facilitated the evolution of traits that enable efficient prey search (e.g., sensory systems, cognitive abilities, and behavioural strategies). Optimal foraging theory, a framework based on optimization models with energy as the primary currency, has been central to understanding how animals maximize their efficiency when searching for and consuming food [1–3]. Many predictions derived from the theory were empirically tested, providing insights into search strategies, food choice, and giving-up times in food patches, among other aspects. Traditionally, these tests used small animals (insects, birds, and fishes) in captivity or in controlled setups (e.g., food presented to wild animals) [1, 2, 4]. Only recently have researchers begun attempting to test these predictions in free-ranging, large aquatic predators (e.g., marine mammals, seabirds, and sharks) using electronic tags [5–11]. However, whether and how they follow the theory’s predictions in the three-dimensional aquatic environment remains poorly understood.

In the theory, an important factor affecting foraging success is behaviour modification based on recent experience [3]. Regarding where central place foragers should concentrate their search efforts, simple behavioural strategies depending on the types of food have been proposed. If food distribution largely remains constant across several consecutive visits, animals would search the same area when prior foraging attempts are successful and move to another area when they are unsuccessful (the ‘win-stay, lose-shift’ strategy) [3, 12]. Conversely, if animals deplete food in one visit, the opposite strategy, ‘win-shift, lose-stay’, might perform best [13]. Moreover, if food distribution is completely unpredictable, animals’ decision-making may be independent of the previous outcome [12]. Given that large aquatic predators typically feed on clustered small prey (e.g., forage fishes, zooplankton), the ‘win-stay, lose-shift’ strategy may be widely employed by these animals.

Another important concept related to the ‘win-stay, lose-shift’ strategy is area-restricted search (ARS), a specific movement pattern observable in animals’ horizontal movement paths [14, 15]. Animals searching for clustered food will likely find multiple food items within a small area. Thus, they are expected to exhibit fast and straight movements in food-poor areas and transition to slow and tortuous movements (that is, ARS) once they locate food. Detecting ARS in animals’ movement paths (e.g., satellite tracking data) is a common approach for identifying regions important to the animal [16]. By assuming that ARS represents foraging behaviour, previous studies have investigated environmental features that facilitate foraging, the spatial scale of prey search, important areas for species conservation, among other aspects [17–19].

Despite being influential concepts, these experience-based behavioural modifications have not been sufficiently tested in large aquatic predators. Although recording their two-dimensional (sometimes three-dimensional) movement paths has become standard with advances in biologging techniques [20], simultaneously recording their movements and foraging success on known prey species remains challenging. Previous studies have analysed satellite tracking data of aquatic predators, combined with estimates of food intake (derived from biologging data or weight measurements), to assess whether variations in movement paths follow the ‘win-stay, lose-shift’ strategy [21–23], and whether ARS patterns emerge in response to prey encounter events [24–27]. These studies have yielded negative or slightly positive support. However, they examined animal foraging behaviour at large spatial scales (> 10 km) without identifying prey species. Given the limited prey detection ranges of predators and the typical scale of prey aggregations (smaller than 100’s of meters), experience-based behavioural modifications may become more evident if the foraging behaviour of large aquatic predators feeding on specific, clustered prey is examined at a finer scale. For example, Antarctic fur seals hunting krill were shown to move shorter linear distances between dives when they appeared to capture more prey (assessed by acceleration signals) during a few prior dives [28].

**Fig. 1:**
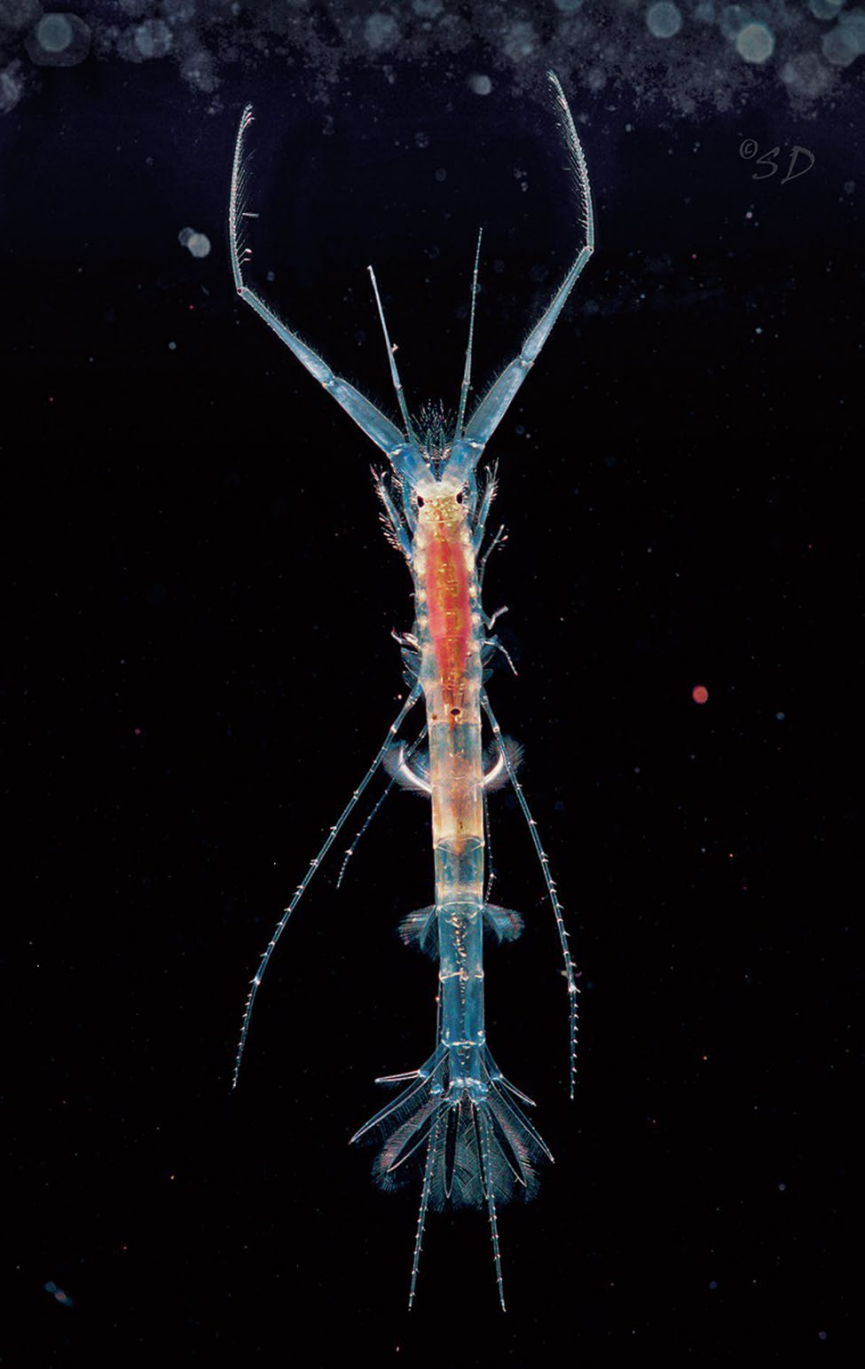
Endemic, planktonic amphipod *Macrohectopus branickii* in Lake Baikal (Photo credit: S. Didorenko)

Baikal seals (*Pusa sibirica*) in Lake Baikal, Russia, provide an excellent model in this respect. Although traditionally considered exclusive fish consumers [29], a recent biologging study [30] and earlier stable-isotope analyses [31] demonstrated that they also hunt the endemic, tiny (< 0.1 g), planktonic amphipods *Macrohectopus branickii* individually (Fig. 1). To make this tiny prey energetically profitable, Baikal seals exhibit the highest foraging rates ever recorded for single-prey-feeding (i.e., non-filter-feeding) aquatic mammals, with over 50 catches per dive and thousands of catches per day [30]. Such high foraging rates can be partly attributed to the enormous abundance, dense patches, and predictable diel vertical migration of *M. branickii* [32–34]. Baikal seals apparently track this diel vertical migration by gradually adjusting their dive depths throughout the night [30, 35]. However, additional behavioural factors, such as experience-based behavioural modification, may also play a role.

In this study, using Baikal seals as a model, we examined whether and how the variations in movement paths relate to recent foraging success in large aquatic predators. We capitalized on our unique dataset where fine-scale diving behaviour of Baikal seals was recorded alongside estimated timings of feeding events on a single planktonic prey species (*M. branickii*) [30]. Moreover, we reconstructed their three-dimensional movement paths during consecutive foraging dives using the dead-reckoning method. By doing so, we tested the hypotheses that (i) Baikal seals would modify three-dimensional movement path (distance, direction) during dives based on the foraging success of prior dives, as expected for the ‘win-stay, lose-shift’ strategy, and (ii) such behavioural modifications would result in ARS patterns when three-dimensional movement paths are projected onto the horizontal plane.

## Methods

Fieldwork was conducted at Ushkany Islands (53.85°N, 108.65°E) in Lake Baikal, Russia, in June 2018 [30]. Briefly, eight seals hauling out onto the shore were manually captured; these included two males and six females, with a mean ± SD body mass of 51.3 ± 11.8 kg. All individuals were adults (at least six years old), as assessed by growth layers in their flipper claws [36]. They were physically restrained and instrumented with a package consisting of a multi-sensor data logger (ORI1300-3MPD3GT, Little Leonardo), a video camera (DVL2000M130-SW or DVL400M, Little Leonardo), a float, a time-scheduled release system (Little Leonardo), a satellite transmitter (SPOT, Wildlife Computers), and a radio transmitter (Advanced Telemetry Systems) before being released into the lake. The package detached 2–4 days after deployment and was located via radio telemetry, then recovered by boat. The data logger recorded depth, temperature, and swim speed at 1 s intervals, and tri-axial acceleration and geomagnetism at 1/20 s intervals throughout the deployment periods. The video camera recorded images at 640 × 480 pixels at 30 frames per second, supplemented by flashing LEDs, for approximately two hours (limited by battery life), and was programmed to start filming after a preset delay.

Data were analysed using the software Igor Pro (WaveMetrics) with the Ethographer extension [37]. A dive was defined as any excursion below the surface to a depth of > 2 m. Pitch of the animals was estimated from low-frequency signals of longitudinal acceleration. Heading (or bearing) was calculated from tri-axial accelerations and tri-axial geomagnetism using the ThreeD_path extension [38]. Swim speed measured by the propeller sensor was calibrated using independent estimates of swim speed based on depth change rates and pitch during periods of steep pitches [39]. Depth, swim speed, pitch, and heading obtained every second allowed us to reconstruct three-dimensional movement paths starting at the known release location of the animals on the shore. However, we acknowledge that location estimates based on this dead-reckoning method are prone to errors due to internal factors (e.g., accumulation of measurement inaccuracies over time) and external factors (e.g., currents) [40]. Therefore, we only examined relative changes in diving locations during each dive bout without converting location estimates into absolute units (longitude and latitude).

**Fig. 2:**
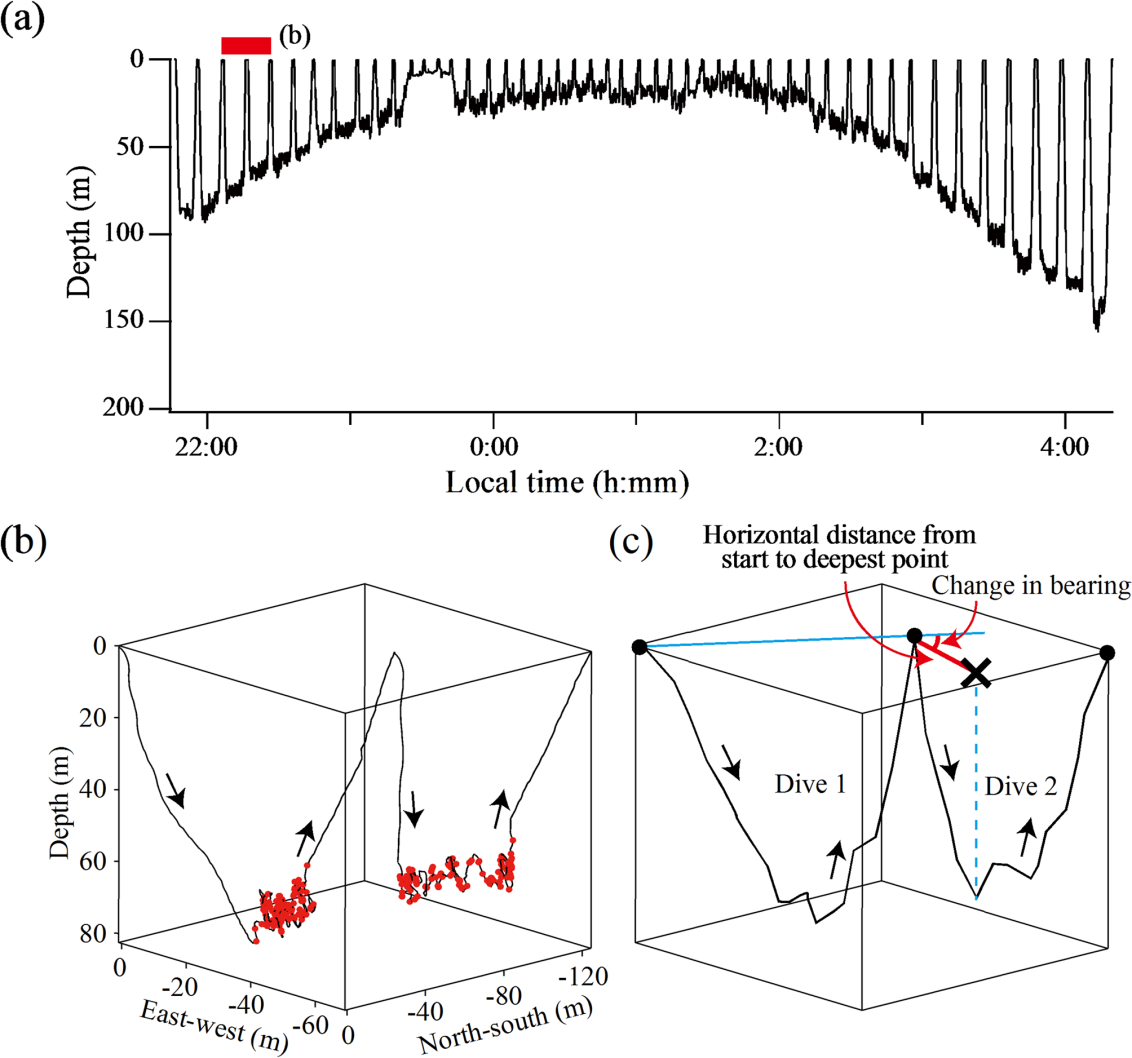
(**a**) Example of an amphipod-feeding dive bout of a Baikal seal recorded during the night. Note the gradual changes in dive depth over time, with shallower dives recorded around midnight (0:00–2:00 local time). The red bar represents two dives detailed in (**b**). (**b**) Three-dimensional movement paths of two consecutive dives, with red dots indicating prey capture events. Horizontal positions are represented by east-west and north-south distances from the initial point, with negative values indicating west and south. (**c**) Simplified illustration of the two dives shown in (**b**), explaining the testing of the ‘win-stay, lose-shift’ strategy. Our working hypotheses are as follows. (i) The horizontal distance from the start to the deepest point of a dive (red line) is negatively related to the foraging success of the prior dive. (ii) The change in bearing between consecutive dives (red angle) is positively related to the foraging success of the prior dive

Foraging dives targeting amphipods (*M. branickii*) were identified by drops in swim speed during the bottom phase, as validated by simultaneous video and behaviour recordings [30]. For hypotheses testing, foraging dive bouts were defined as those with more than four consecutive dives (Fig. 2a). A small number of dives (33 out of 972) in the bouts with unusual characteristics (e.g., exceptionally shallow or short dives, dives lacking clear drops in swim speed) were visually identified and excluded from the analyses. For each dive, the number of prey capture events was estimated based on the high-pass filtered vectorial sum of triaxial accelerations [(x^2^ + y^2^ + z^2^)^0.5^] (Fig. 2b). This method was validated using video footage showing feeding events, providing a high coefficient of determination (R^2^ = 0.94) [30]. If seals employ the ‘win-stay, lose-shift’ strategy at a dive-to-dive scale, they would move shorter horizontal distances and exhibit larger changes in bearing (i.e., more tortuous movements) following more successful foraging dives (Fig. 2c). We regarded the location (on the horizontal plane) where the greatest depth was reached during a dive (marked with an X symbol in Fig. 2c) as the representative ‘destination’ of the animals during that dive. Our two hypotheses were as follows. (i) Horizontal distance from the start to the deepest point of a dive is negatively related to the number of prey capture events in the prior dive. (ii) Changes in bearing between two consecutive dives (i.e., the absolute difference in angle between the horizontal line connecting the start and deepest point of a dive and that connecting the start and endpoint of the prior dive) are positively related to the number of prey capture events in the prior dive (Fig. 2c). These hypotheses were tested using linear mixed-effect models with seal ID as a random factor, implemented in R using the lme4 package [41], with statistical significance set at *P* < 0.05. Correlations were also tested for individual seals using linear models. To examine the robustness of our analyses, we also set a different ‘destination’ during dives—the location (on the horizontal plane) where the initial prey capture event was observed—and repeated the analyses.

Horizontal movement paths during amphipod-feeding dive bouts were visualized by plotting the location of each dive represented by the middle-distance point between the start and end locations. For each path, the straightness index was calculated as the ratio of the net displacement (i.e., straight-line distance between start and end locations of the bout) to the total travelled length (i.e., the sum of straight-line distances between consecutive dives). It can range from 0 (moving back to the origin) to 1 (a perfectly straight track).

## Results

From a total of 556 h of behavioural records obtained from eight seals, 42 foraging dive bouts targeting *M. branickii*, composed of 939 individual dives, were detected. The dive bouts primarily occurred at night, with a gradual decrease and increase in dive depth before and after midnight, respectively (Fig. 2a), as previously reported [30, 35]. The reconstructed three-dimensional movements paths showed that seals exhibited complex movements, including upward and downward swimming and turns, while feeding primarily during the bottom phase of dives (Fig. 2b).

**Fig. 3:**
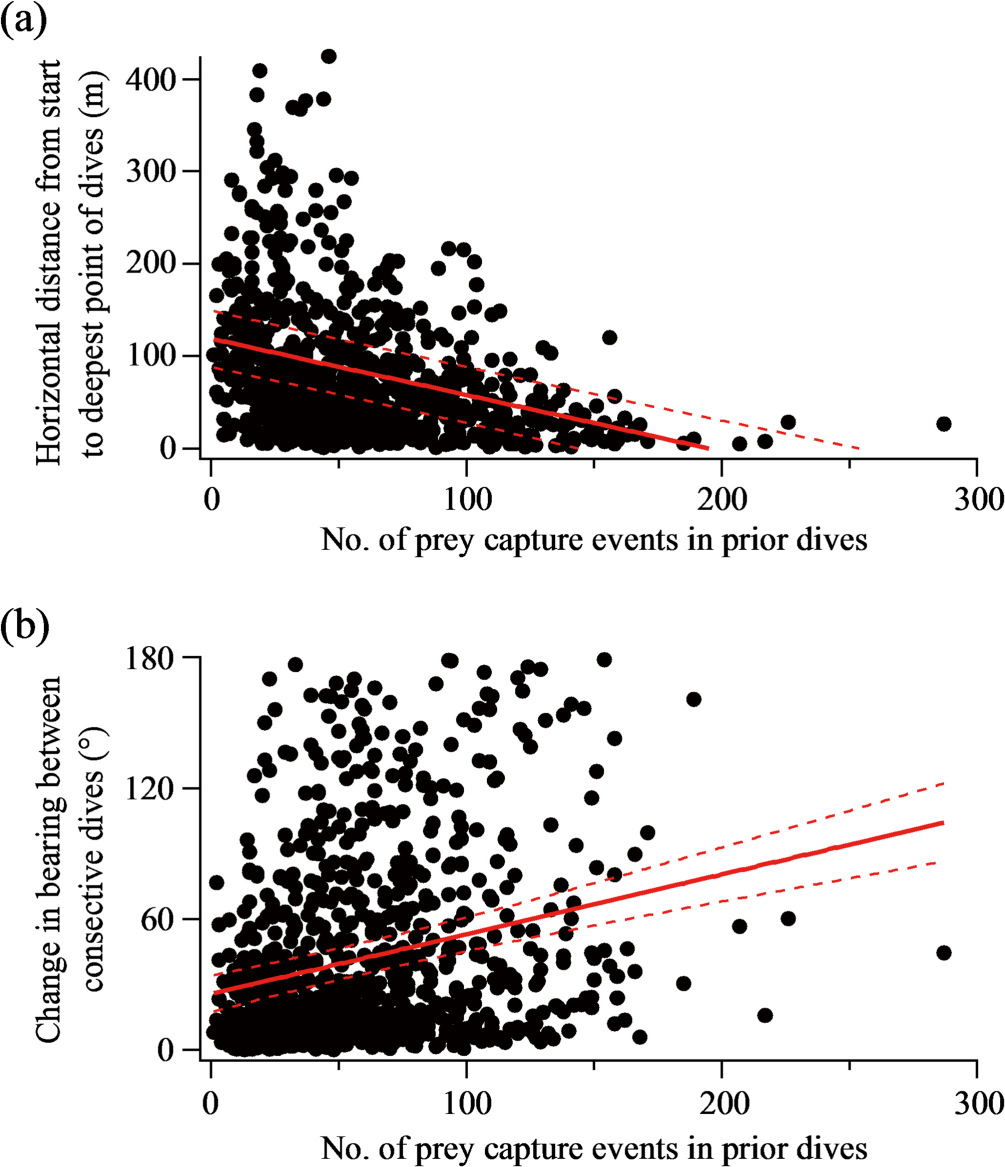
(**a**) Horizontal distance from the start to the deepest point of dives and (**b**) change in bearing between consecutive dives, as a function of the number of prey capture events in prior dives. Data pooled from all eight individual seals. Red lines represent regression lines from linear mixed-effect models (*Y*=-0.61×*X* + 118.7 in panel a and *Y* = 0.27×*X* + 25.6 in panel b), with dashed lines indicating 95% confidence intervals

The horizontal distance from the start to the deepest point of a dive (Fig. 2c) decreased with the number of prey capture events in the prior dive in the pooled data including all eight individual seals (*P* < 0.0001) (Fig. 3a). When data for individual seals were analysed separately, statistically significant negative relationships were found in all eight individuals (Fig. S1). Changes in bearing between two consecutive dives (Fig. 2c) increased with the number of prey capture events in the prior dive in the pooled data (*P* < 0.0001), despite greater variability than in horizontal distance for a given level of foraging success of prior dives (Fig. 3b). When data for individual seals were analysed separately, statistically significant positive relationships were found for five out of the eight individual seals (Fig. S2). Seal ID (as a random factor) explained 35% and 5% of the variance in the pooled models of horizontal distance (Fig. 3a) and changes in bearing (Fig. 3b), respectively. Changing our definition of the ‘destination’ of each dive—from the deepest point to the initial prey capture point—yielded similar results. The horizontal distance decreased (*P* < 0.0001), while changes in bearing between two consecutive dives increased (*P* < 0.0001), with the number of prey capture events in the prior dive (Fig. S3).

**Fig. 4:**
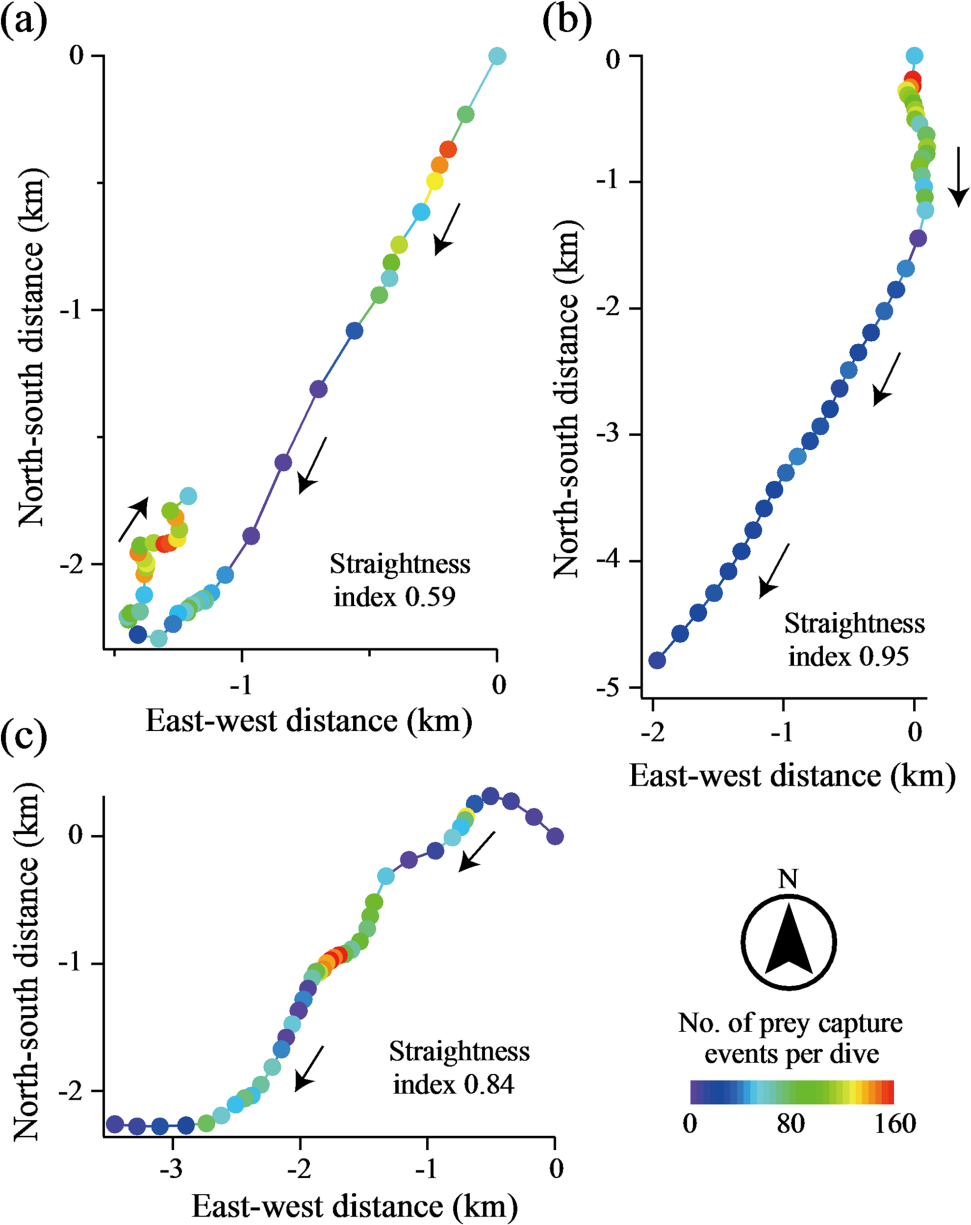
Three examples, taken from different individuals, showing changes in diving location during amphipod-feeding dive bouts. Each circle represents a dive, with the number of prey capture events indicated by colour. Locations are plotted by east-west (x-axis) and north-south distances (y-axis) from the location of the initial dive, with negative values indicating west and south. The straightness index is provided for each dive bout. Note that dives with high foraging success are associated with shorter dive-to-dive distances, larger changes in bearing, or both. Dive bout in (**a**) is the same as that shown in Fig. 2 (a), displaying depth data

On the horizontal plane, seals generally exhibited directional movement paths with a mean ± SD straightness index of 0.80 ± 0.23 (*N* = 42 dive bouts) during foraging dive bouts, although turns and curves were also observed (Fig. 4). Dives with high foraging success were associated with shorter dive-to-dive distances, larger changes in bearing (i.e., more tortuous movements), or both. This result aligns with the negative and positive effects, respectively, of the foraging success of prior dives on horizontal distance and changes in bearing of subsequent dives (Fig. 3).

## Discussion

### Win-stay, lose-shift strategy

We found that dive-to-dive decision-making by Baikal seals feeding on *M. branickii*, a clustered planktonic prey, follows the predictions of the ‘win-stay, lose-shift’ strategy, although they continuously move and never ‘stay’ at a location in the strict sense. When the prior dive was more successful, Baikal seals moved shorter horizontal distances during the search phase of the next dive (Fig. 3a). Antarctic fur seals hunting clustered krill behave similarly [28]. Remarkably, the trend we observed was evident not only in the pooled data but also in all eight individual datasets (Fig. S1). Thus, the effect of recent foraging success on horizontal swimming distances is strong and consistent across individuals. Baikal seals also exhibited larger changes in bearing when the prior dive was more successful (Fig. 3b). This trend was weaker compared to that for horizontal distances, as evidenced by greater variability in the pooled data for a given level of foraging success (Fig. 3) and by the lack of statistically significant trends in three of the eight individual datasets (Fig. S2). The weaker trend in changes in bearing is not surprising, because to exploit the same patch after successful visits, animals need to restrict their horizontal movements but do not necessarily need to change direction. Our results are robust to the definition of ‘destination’ during dives, as shown by additional analyses where the destination was set at the initial prey capture point, rather than the deepest point, during each dive (Fig. S3). Overall, our results provide strong evidence that Baikal seals make decisions regarding whether they stay or move further depending on recent foraging success. The among-individual variation we observed may stem from age differences (e.g., older individuals may exhibit greater behavioural flexibility); however, we were unable to test this possibility due to the lack of precise age estimates.

Most previous studies that tested the ‘win-stay, lose-shift’ strategy for large aquatic predators found only weak support [21–23], except for the Antarctic fur seal study [28]. There are several possible reasons why we detected strong effects. First, we examined movement paths across dives based on the dead-reckoning method at a spatial scale of tens to hundreds of meters (Fig. 2b). In contrast, previous studies examined movement paths across foraging trips using satellite tags (Argos transmitters or GPS loggers) at scales of tens to hundreds of kilometres [21–23]. At such large scales, geophysical factors (e.g., seafloor topography) and oceanographic features (e.g., currents) within animal movement ranges are highly heterogeneous, which could potentially mask experience-based behavioural modifications of predators. Second, we examined the behaviour of Baikal seals hunting clustered planktonic prey (*M. branickii*). The ‘win-stay, lose-shift’ strategy theoretically performs best in predictable habitats [12]. This condition is largely met in the seal-amphipod system, at least within our study area (middle Baikal) and period (summer), as *M. branickii* form large aggregations and exhibit diel vertical migrations [32, 34], which are tracked by Baikal seals (Fig. 2a). In contrast, the previous studies referenced above examined movement paths where predators captured unidentified prey, likely comprising multiple species. Therefore, experience-based behavioural modifications might have been obscured by prey characteristics (e.g., abundance, mobility, and patchiness) and their variations across multiple species.

Is the pattern we observed in Baikal seals representative of other large aquatic predators? Several penguin species descend at steeper pitches when they spend longer periods or exhibit more small-scale vertical movements (indicative of foraging efforts) during the bottom phase of the prior dive [42, 43]. Similarly, southern elephant seals [44] and humpback whales [45] increase descent and ascent angles when they appear to encounter more prey during dives, as indicated by body acceleration in seals and the frequency of lunge-feeding behaviour in whales. These observations, consistent with the ‘win-stay, lose-shift’ strategy, suggest that experience-based behavioural modifications at a dive-to-dive scale may be common among large aquatic predators feeding on clustered prey, regardless of whether they are single-prey feeders (e.g., penguins, seals) or filter feeders (e.g., baleen whales). If this strategy can be applied to broader spatiotemporal scales, it may help explain some of the seasonal movements observed in large aquatic predators. For example, migration tracks of krill-eating blue whales tend to follow the long-term average (across past years) of seasonal phytoplankton blooms—a proxy for krill availability—rather than contemporaneous patterns [46]. Understanding the roles of memory and experience in shaping foraging behaviour across scales in dynamic aquatic environments remains a key challenge in movement ecology.

### Area restricted search (ARS)

As hypothesized, the ‘win-stay, lose-shift’ strategy exhibited by Baikal seals at a dive-to-dive scale resulted in horizontal movement paths of dive bouts closely resembling ARS. The paths were predominantly directional, and dives with high foraging success were associated with subsequent decreases in speed, increases in tortuosity, or both (Fig. 4). Our empirical data support the association of ARS with foraging success in a large aquatic predator. Previous studies that examined such associations using animal movement paths recorded by satellite tags and proxies of foraging activity found negative or only weak support [24–27]. These studies suggested that (i) movement paths associated with foraging cannot be accurately identified by satellite (e.g., Argos) tracking data due to relatively large errors [25], and (ii) animals foraging over oceanic habitats do not always move in ways predicted by simple search theory [24, 26, 27]. Interestingly, northern elephant seals exhibit tortuous movements in three-dimensional space (called volume-restricted search behaviour) during dives, which are associated with foraging events inferred from accelerometers attached to the mandibles [5]. Dolphins spend longer periods within detection ranges of stationary, passive acoustic monitoring systems when foraging activity (detected by echolocation clicks) was high at the beginning, supporting the association of ARS with foraging success [47]. Taken together, large aquatic predators do exhibit foraging-associated ARS, but it is most evident when their movements are tracked at a dive-to-dive scale with high resolution. Moreover, given that ARS is inherently linked to the ‘win-stay, lose-shift’ strategy, the types of prey also play a role. In the predator-prey system we studied, the dense patches and predictable distributions of *M. branickii* might induce Baikal seals to exhibit clear ARS.

Our findings have important implications for the tracking studies of large aquatic predators. An increasing number of studies utilize satellite tags to record their horizontal movement paths, offering valuable insights into their habitat utilization and contributing to conservation efforts in dynamic ocean environments [48, 49]. Many of these studies lack direct information on foraging and instead examine the presence, scale, timing, and location of ARS, assuming that ARS represents foraging behaviour [17–19]. Our findings, combined with previous efforts, show that the correctness of this assumption depends on the scale of measurements and the types of prey. To validate the underlying assumption of ARS, fine-scale measurements of animals’ movement paths alongside the detection of foraging events on known prey species are needed, as demonstrated in this study. An important consideration here is that foraging movements are influenced not only by prey availability but also by morphological and physiological constraints, as evidenced by the mismatch between predators’ swimming depth and prey depth in filter-feeding whale sharks [50].

### Foraging ecology of Baikal seals

Lastly, our results suggest that experience-based behavioural modifications are key for Baikal seals to sustain their unusually high foraging rates on *M. branickii*, exceeding 50 catches per dive [30]. Coupled with gradual changes in dive depth following the diel vertical migration of prey, adjustments in swimming distance and direction based on prior foraging success enable Baikal seals to efficiently exploit dynamic patches of *M. branickii* in three-dimensional space. *M. branickii* is extremely abundant, with an estimated biomass from hydroacoustic monitoring for Barguzin Bay (close to our study area) of 3,700–3,800 tons over a 407–426 km^2^ area [34]. Its biomass over the entire lake was estimated to be 110,000 tons [33]. Additionally, Baikal seals possess the most specialized ‘comb-like’ post-canine teeth among northern phocid species [30, 51]. This tooth shape allows the animals to expel water while retaining prey in their mouths after each hunting action, thereby sustaining high foraging rates without excessive water intake [30]. Taken together, these behavioural, environmental, and morphological factors contribute to the unique predator-prey system of Baikal seals and *M. branickii*, underpinning an exceptionally high predator-prey body mass ratio (> 500,000) for a predator hunting single prey at a time. However, Baikal seals also feed on fishes such as energy-rich, pelagic sculpins (*Comephorus baicalensis* and *C. dybowskii*) [29]. Whether experience-based behavioural modifications can be observed when feeding on fishes, which are more mobile and less clustered than *M. branickii*, remains unknown. Furthermore, investigating the environmental and internal factors that drive Baikal seals to switch between energy-rich fish and tiny, clustered amphipods would be fruitful in future studies, particularly within the framework of optimal diet and patch use [2].

## Conclusions

We examined the fine-scale, three-dimensional diving behaviour of Baikal seals feeding on clustered planktonic prey (*M. branickii*). Consistent with the ‘win-stay, lose-shift’ strategy, they moved shorter horizontal distances and exhibited greater changes in bearing when prior dives were more successful. Their horizontal movement paths were predominantly directional, with successful foraging dives associated with subsequent decreases in speed and increases in tortuosity, characteristics expected from the concept of ARS. Our study provides strong support for behavioural modifications based on recent foraging success, contrasting with previous studies that focused on the large-scale movement paths of aquatic predators feeding on unidentified prey. Optimal patch use appears especially important for Baikal seals and other large aquatic predators [52] that feed on disproportionately small prey. The research demonstrated here can be extended to other aquatic predators and prey types, leading to a better understanding of the roles of memory and experience in shaping foraging behaviour, as well as more accurate interpretation of simpler animal tracking data where direct foraging information is lacking.

## Supplementary Information

Below is the link to the electronic supplementary material.

Supplementary Material (Figs. S1-3)

## Data Availability

The datasets used in this study are available from the corresponding author upon reasonable request.
