# Supplementary material for: Experience-based optimal foraging on planktonic prey in Baikal seals
Source: Mov Ecol. 2025 Sep 30;13:65. doi: 10.1186/s40462-025-00593-y (PMC12487204; doi:10.1186/s40462-025-00593-y)
Supplement: Supplementary file 1 — Supplementary Material (Figs. S1-3) [file 40462_2025_593_MOESM1_ESM.docx]

**Fig. S1.** Horizontal distance from the start to the deepest point of dives as a function of the number of prey capture events in prior dives, shown for individual seals. Statistically significant negative relationships were found for all eight individuals, as indicated by red regression lines and dashed 95% confidence intervals.

**Fig. S2.** Change in bearing between consecutive dives as a function of the number of prey capture events in prior dives, shown for individual seals. Statistically significant positive relationships were found for five of the eight individuals, as indicated by red regression lines and dashed 95% confidence intervals.

**Fig. S3.** (a) Horizontal distance from the start to the initial prey capture point of dives and (b) change in bearing between consecutive dives, as a function of the number of prey capture events in prior dives. Data pooled from all eight individual seals. Red lines represent regression lines from linear mixed-effect models (*Y*=-0.26×*X*+52.6 in panel a and *Y*=0.35×*X*+25.5 in panel b), with dashed lines indicating 95% confidence intervals.
